# Mechanism of Unusual Isosymmetric Order-Disorder Phase Transition in [Dimethylhydrazinium]Mn(HCOO)_3_ Hybrid Perovskite Probed by Vibrational Spectroscopy

**DOI:** 10.3390/ma14143984

**Published:** 2021-07-16

**Authors:** Jan Albert Zienkiewicz, Edyta Kucharska, Maciej Ptak

**Affiliations:** 1 Institute of Low Temperature and Structure Research, Polish Academy of Sciences, Okólna 2 Str., 50-422 Wrocław, Poland; j.zienkiewicz@intibs.pl; 2 Department of Bioorganic Chemistry, Faculty of Production Engineering, Wroclaw University of Economics and Business, Komandorska 118/120 Str., 53-345 Wrocław, Poland; edyta.kucharska@ue.wroc.pl

**Keywords:** hybrid perovskite, phase transition, order-disorder, dimethylhydrazinium cation

## Abstract

[DMHy]Mn(HCOO)_3_ (DMHy^+^ = dimethylhydrazinium cation) is an example of an organic–inorganic hybrid adopting perovskite-like architecture with the largest organic cation used so far in the synthesis of formate-based hybrids. This compound undergoes an unusual isosymmetric phase transition at 240 K on heating. The mechanism of this phase transition has a complex nature and is mainly driven by the ordering of DMHy^+^ cations and accompanied by a significant distortion of the metal–formate framework in the low temperature (LT) phase. In this work, the Density Functional Theory (DFT) calculations and factor group analysis are combined with experimental temperature-dependent IR and Raman studies to unequivocally assign the observed vibrational modes and shed light on the details of the occurring structural changes. The spectroscopic data show that this first-order phase transition has a highly dynamic nature, which is a result of balanced interplay combining re-arrangement of the hydrogen bonds and ordering of DMHy^+^ cations. The tight confinement of organic cations forces simultaneous steric deformation of formate ions and the MnO_6_ octahedra.

## 1. Introduction

3D hybrid metal–formate perovskites, a class of multifunctional materials described by the general formula [A]M^II^(HCOO)_3_, where A is an ammonium cation and M^II^ denotes a divalent metal cation, caught the attention of materials scientists in a few recent years because of their unusual luminescent, [1,2] ferroelastic, [3,4] ferroelectric, [5,6,7] dielectric, [2,4,8] magnetic [2,9,10] or multiferroic [3,11,12,13] properties. These properties originate from order-disorder phase transitions (PTs) that enable utilizing them as molecular switches [14,15,16].

Up to date, hydrazine and its derivatives were used only a few times as templating agents in the synthesis of formate-based hybrids. Since the hydrazinium cation (Hy^+^) has a small size, [Hy]M^II^(HCOO)_3_ compounds with M^II^ = Mn^2+^, Zn^2+^ and Fe^2+^ can adopt two types of structure, namely 4^12^·6^3^ perovskite-like with cubic cavities or 4^9^·6^3^ chiral with hexagonal channels [17,18,19]. Analogues comprising Co^2+^ and Mg^2+^ ions were found to adopt only chiral architecture. [Hy]M^II^(HCOO)_3_ perovskites undergo an order-disorder PT near 350 K from the LT ferroelectric *Pna*2_1_ to the high-temperature (HT) paraelectric *Pnma* phase. However, chiral analogues transform from the ferroelectric *P*2_1_2_1_2_1_ LT to the ferroelectric *P*6_3_ HT phase in the 336–380 K range. The chiral [Hy]Mn(HCOO)_3_ adopts exceptionally the *P*2_1_ LT symmetry and exhibits lower PT temperature, 296 K [17,18,19].

The increased size of the methylhydrazinium cation (MHy^+^) affects the crystal structure, and only perovskite-like architecture is preferred for [MHy]M^II^(HCOO)_3_ (M^II^ = Mn^2+^, Mg^2+^, Fe^2+^, Zn^2+^) [20]. All MHy^+^ analogues experience two PTs. The first one occurs in the 168–243 K range from the LT polar phase of *P*1 symmetry to the intermediate polar *R*3*c* phase, whereas the second one, near 310–327 K, to the paraelectric HT *R*$\bar{3}$*c* phase [20].

Further increase in the cation size caused by the presence of the second methyl group leads to complete suppression of non-centrosymmetric structures in [DMHy]Mn(HCOO)_3_, (DMHy^+^ denotes 1,1-dimethylhydrazinium cation). This crystal, recently synthesized by us, exhibits unusual order-disorder PT among metal–formate hybrids [2]. In fact, [DMHy]Mn(HCOO)_3_ is the first example of a formate-based perovskite that does not change the space group symmetry as a result of LT ordering. Therefore, it is of great importance to elucidate the mechanism of this PT in detail and focus on the hydrogen bond (HB) interactions. Furthermore, DMHy^+^ cation is the largest organic cation successfully used in the synthesis of formate-based hybrids. The tolerance factor of [DMHy]Mn(HCOO)_3_ perovskite is at the theoretically predicted limit by Kieslich et al. [21], making this compound an interesting model for structure-stability considerations.

It is well-known that vibrational spectroscopy, as a support for X-ray diffraction methods, is commonly used to shed some light on the dynamical properties and mechanisms of PTs occurring in hybrid organic–inorganic perovskites. A great advantage of Raman and IR spectroscopy is its high sensitivity to local structural changes involving dynamics of light atoms that are responsible for the formation of HBs. Since the arrangement of HBs is usually strongly affected during PTs, detailed studies of phonon properties can give access to structural information not available using other probing techniques.

IR and Raman studies were used to understand PT mechanisms observed in Hy^+^ and MHy^+^ analogues [18,20]. Therefore, we have decided to undertake similar studies for [DMHy]Mn(HCOO)_3_ and compare its phonon properties to former compounds. Detailed temperature-dependent studies with small temperature increments allowed to obtain deeper insight into structural changes occurring in this compound at 244.4 K (283.0 K) on cooling (heating) [2] and to elucidate the main driving force of the PT. It is worth adding that the *P*2_1_/*n*–*P*2_1_/*n* isosymmetric PT is also interesting from the spectroscopic point of view because weak changes of vibrational selection rules are expected in spite of significant structural changes and the associated high change of entropy [2]. Moreover, the isosymmetric and isostructural PTs occurring in the coordination polymers are still poorly understood [16,22,23,24,25,26,27,28,29].

The main goal of this paper is to analyse the phonon properties of [DMHy]Mn(HCOO)_3_ as a function of temperature. We propose an assignment of the observed IR and Raman bands based on literature data for Hy^+^ and MHy^+^ analogues supported by DFT calculations reported in this paper for DMHy^+^ cation. We show that the presence of an additional methyl group significantly affects the phonon properties of [DMHy]Mn(HCOO)_3_. In the discussion of temperature-dependent spectra, particular attention is paid to the factor group analysis in order to obtain deep insight into the PT mechanism.

## 2. Materials and Methods

### 2.1. Materials and Synthesis

The 1,1-dimethylhydrazine (Sigma-Aldrich, Saint Louis, MO, USA), formic acid (85%, Avantor Performance Materials Poland, Gliwice, Poland), anhydrous methanol (Sigma-Aldrich, Saint Louis, MO, USA) and manganese(II) perchlorate hexahydrate (Sigma-Aldrich, Saint Louis, MO, USA) were purchased from commercial sources and used without further purification.

The cubic transparent crystals of [DMHy]Mn(HCOO)_3_ were obtained from a sealed and undisturbed mixture of two solutions. The first containing 40 mmol (ca. 3 mL) of 1,1-dimethylhydrazine dissolved in 10 mL of methanol with the addition of 160 mmol (7.25 mL) of HCOOH and the second, composed of 1 mmol (0.3619 g) of Mn(ClO_4_)_2_·6H_2_O dissolved in 10 mL of methanol. Further details can be found in [2].

### 2.2. Raman ad IR Spectroscopy

A room-temperature (RT) Raman spectrum of the polycrystalline sample was measured in the 4000–75 cm^−1^ range using a FT 100/S spectrometer with YAG:Nd laser excitation at 1064 nm (Bruker, Billerica, MA, USA). The temperature-dependent (80–400 K) Raman spectra of a randomly oriented single crystal in the 3500–50 cm^−1^ range were measured using a Renishaw inVia Raman spectrometer (Renishaw, Wotton-under-Edge, UK), equipped with confocal DM2500 Leica optical microscope, a thermoelectrically cooled CCD as a detector and an Ar^+^ ion laser operating at 488 nm. The temperature was controlled using a THMS600 stage (Linkam Scientific Instruments Ltd., Epsom, Tadworth, UK).

An RT polycrystalline IR spectrum in the range of 4000–400 cm^−1^ (mid-IR) was measured using a Nicolet iS50 infrared spectrometer (Thermo Fisher Scientific, Waltham, MA, USA) as a suspension in nujol (mineral oil) and Fluorolube (Sigma-Aldrich, Saint Louis, MO, USA). Additional mid-IR spectrum was recorded using an ATR module and diamond crystal. A far-IR spectrum in the range of 650–50 cm^−1^ was measured on a polyethylene plate as a suspension in nujol. The temperature-dependent (80–400 K) IR spectra in the 4000–650 cm^−1^ range were measured using a Nicolet iN10 infrared microscope (Thermo Fisher Scientific, Waltham, MA, USA). The temperature was controlled using a THMS600 stage equipped with ZnSe windows (Linkam Scientific Instruments Ltd., Epsom, Tadworth, UK).

### 2.3. Quantum Chemical Calculations

The geometry optimization of the molecular structure of dimethylhydrazine molecule (DMHy) and DMHy^+^ cation was performed using a Gaussian 03 package [30]. All calculations were carried out using density functional three-parameters hybrid (B3LYP) methods [31,32,33] with the 6-311G(2d,2p) [34,35] basis set starting from the X-ray geometry taken from [2]. The harmonic and anharmonic vibrational wavenumbers were also calculated. The calculated harmonic frequencies were scaled using scaling factors (0.96 and 0.98) to correct the evaluated wavenumbers for vibrational anharmonicity and deficiencies inherent to the used computational level. The potential energy distribution (PED) of the normal modes among the respective internal coordinates was calculated for studied compounds using the BALGA program [36]. The data from DFT calculations were input into the BALGA program. The theoretical Raman intensities were calculated using the Chemcraft program [37] that was also used for the visualization of molecules.

## 3. Results

### 3.1. Crystal Structure and Geometry Optimization

**Figure 1:**
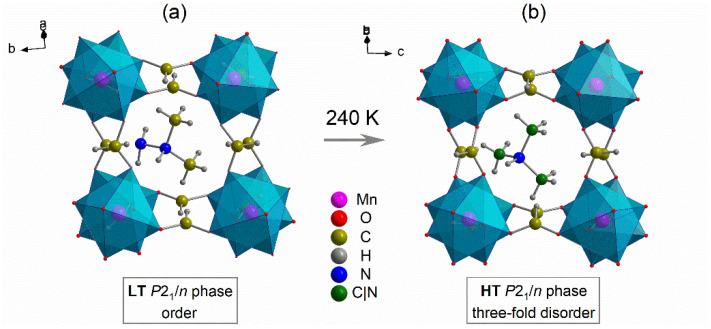
The crystal structure of the LT (**a**) and HT (**b**) phase of [DMHy]Mn(HCOO)_3_.

Both LT and HT phases of [DMHy]Mn(HCOO)_3_ are described by the *P*2_1_/*n* monoclinic symmetry [2]. The crystal structure is built from the manganese–formate 3D framework forming pseudo-cubic voids that accommodate the DMHy^+^. Organic cations balance the negative charge of the manganese–formate framework and are bonded by medium strength HBs. In the HT phase, DMHy^+^ cations exhibit a threefold disorder, while in the LT phase, disorder is no longer observed (Figure 1). In the LT phase, the Mn^2+^ centres occupy only one C_1_ (4e) site, whereas, in the HT phase, they are distributed equally into two C_s_ sites (2a and 2d) [2]. All remaining atoms in both phases occupy C_1_ sites (4e).

The results of geometry optimisation performed for DMHy^+^ and DMHy are presented in Table S1. Figure S1 presents the numbering of atoms used for calculations. The calculated skeletal N1–N4, N4–C5 and N4–C10 distances (1.450, 1.504 and 1.503 Å, respectively) for DMHy^+^ are in good agreement with experimental values obtained using X-ray diffraction methods, i.e., 1.428(6)–1.482(6), 1.398(10)–1.538(8) and 1.476(10)–1.538(8) Å at 300 K and 1.447(3), 1.488(4) and 1.490(4) at 100 K, respectively [2]. The calculated N1–N4–C5, N1–N4–C10 and C5–N4–C10 angles for DMHy^+^ are equal to 115.46, 108.69 and 112.70°, respectively, and correspond well to ranges of values obtained for crystal structures solved at 100 K (109.0(2)–114.2(2)°) [2]. The calculated lengths of C–H and N–H bonds are higher in comparison to the experimental ones, but this is an expected effect caused by imprecise positioning of H atoms by crystallographic methods and by in vacuo character of performing calculations.

The optimised geometry of the DMHy molecule is similar to DMHy^+^. However, the N–C distances seem to be more sensitive than the N–N one to the presence of proton bonded to N4. The lack of proton causes the shortening of N–C and N–N bonds by 3.2% and 1.3%, respectively.

### 3.2. Selection Rules and Factor Group Analysis

All 30 vibrational degrees of freedom for DMHy can be subdivided into 11 stretching and 19 bending modes. The stretching (ν) modes can be roughly described as 4 × ν_as_CH_3_ (antisymmetric), 2 × ν_s_CH_3_ (symmetric), ν_as_NH_2_, ν_s_NH_2_, ν_as_CNC, ν_s_CNC and νNN. The deformational modes can be described as 4 × δ_as_CH_3_, 2 × δ_s_CH_3_, 4 × ρCH_3_ (rocking) and 2 × τCH_3_ (twisting), δNH_2_ (bending), ρNH_2_, ωNH_2_(wagging), τNH_2_, 2 × δCNN and δCNC.

The additional proton bonded to the N4 atom in DMHy^+^ increases the number of vibrational degrees of freedom to 33. Apart from the vibrations mentioned above, there are three additional vibrations involving the N4-H^+^ group, namely νNH^+^, δNH^+^ (in-plane) and γNH^+^ (out-of-plane bending).

IR and Raman spectra of [DMHy]Mn(HCOO)_3_ can be understood by subdividing all Brillouin zone-centre vibrations into internal and external (lattice vibrations). The six internal vibrations of free formate ion (see Table S2) are described as νCH (ν_1_), ν_s_CO (ν_2_) (symmetric stretching), ν_as_CO (ν_4_) (antisymmetric stretching), δOCO (ν_3_), δCH (ν_5_) and γCH (ν_6_) [38]. The 12 formate ions in the primitive cell of [DMHy]Mn(HCOO)_3_ give rise to 72 internal modes (18A_g_ + 18A_u_ + 18B_g_ + 18B_u_) in both the LT and HT phases. The number of expected translational (T’) and librational (L) modes of formate ions is 36 each (9A_g_ + 9A_u_ + 9B_g_ + 9B_u_).

A free DMHy^+^ cation has *C_s_* symmetry similar to isopropylamine [39], and therefore, the symmetries of particular vibrations can be derived (see Table S2). Thus, the 18A′ + 15A″ normal vibrations exhibit factor group splitting to 132 modes (33A_g_ + 33A_u_ + 33B_g_ + 33B_u_) in the studied crystal. The translations and librations of DMHy^+^ give rise to 12 modes each (3A_g_ + 3A_u_ + 3B_g_ + 3B_u_). Although the DMHy^+^ cations are disordered in the HT phase, the total number of their theoretically predicted modes do not change during PT.

The symmetry of Mn^2+^ translations is different for both phases because of occupied sites. In the LT (ordered) phase, the number of expected translations is 12 and distributed into 3A_g_ + 3A_u_ + 3B_g_ + 3B_u_. In the HT phase, the total number is unchanged, however, is distributed into 6A_u_ + 6B_u_ modes. In both phases, three of these translational modes (A_u_ + 2B_u_) are acoustic, thus cannot be detected using IR and Raman spectroscopy.

To conclude, the total number of expected optical modes is 309 (75A_g_ + 80A_u_ + 75B_g_ + 79B_u_) in the HT phase, as well as in the LT phase (78A_g_ + 77A_u_ + 78B_g_ + 76B_u_). All g-symmetry modes are Raman-active, and u-symmetry modes are IR-active. Therefore, the number of expected Raman and IR bands is 150 (75A_g_ + 75B_g_) and 159 (80A_u_ + 79B_u_) in the HT phase, respectively, and 156 (78A_g_ + 78B_g_) and 153 (77A_u_ + 76B_u_) in the LT phase, respectively. It should be added that because u-symmetry modes are solely IR-active, the T’(Mn^2+^) is not detectable in the Raman spectrum of the HT phase.

### 3.3. DFT Calculations

The calculated wavenumbers, together with PEDs, are listed in Table S3. The theoretical spectra calculated in harmonic and anharmonic approximations are presented in Figure S2. The results of DFT calculations for DMHy showed that 21 vibrational modes have a nearly pure (96% and higher) contribution of a single vibration, 4 modes have the main contribution with PED ranging from 71% to 81%, and the remaining 5 modes have more complex origin. The protonation causes stronger coupling of observed modes, i.e., 15 bands have close to pure contribution (95% or higher), 13 bands have a clearly dominant contribution (63–89%), and 3 bands exhibit stronger coupling. Furthermore, the protonation-induced shifts of some bands are evidenced. For instance, the strongest downshifts in harmonic approximation are observed for the ν_s_NH_2_ (by 125 cm^−1^) and ν_s_CH_3_ (by 163–169 cm^−1^) modes. This is an interesting behaviour since their antisymmetric counterparts are less sensitive and downshifted only by 24 and 61–106 cm^−1^.

The largest differences between harmonic and anharmonic wavenumbers are observed for both DMHy and DMHy^+^ for bands originating from the νNH_2_ and νCH_3_ vibrations. Interestingly, anharmonicity in DMHy is stronger for ν_s_CH_3_ (downshifts up to 182 cm^−1^) than for ν_as_CH_3_ (downshifts in the 125–153 cm^−1^ range) and comparable for ν_s_NH_2_ (downshifts by 197 cm^−1^) and ν_as_NH_2_ (downshift by 188 cm^−1^) counterparts. For DMHy^+^, the tendency observed for νCH_3_ is opposite, namely ν_s_CH_3_ and ν_as_CH_3_ are downshifted by 100–103 and 143–144 cm^−1^, respectively. Among νNH_2_ vibrations, the stronger anharmonicity is observed for ν_as_NH_2_ (downshift by 167 cm^−1^) and for ν_s_NH_2_ (downshift by 107 cm^−1^). The anharmonicity of νNH^+^ is comparable to that observed for ν_as_NH_2_ and reaches a value of 157 cm^−1^. Furthermore, τNH_2_ and τCH_3_ vibrations of DMHy exhibit negative anharmonic shifts (from −18 to −40 cm^−1^). This effect is not evidenced for DMHy^+^; therefore, the high sensitivity of bands assigned to νNH_2_, νCH_3_, τNH_2_ and τCH_3_ to the protonation may be related to their stronger intrinsic anharmonicity.

### 3.4. Room-Temperature IR and Raman Spectra and Assignment of Bands

**Figure 2:**
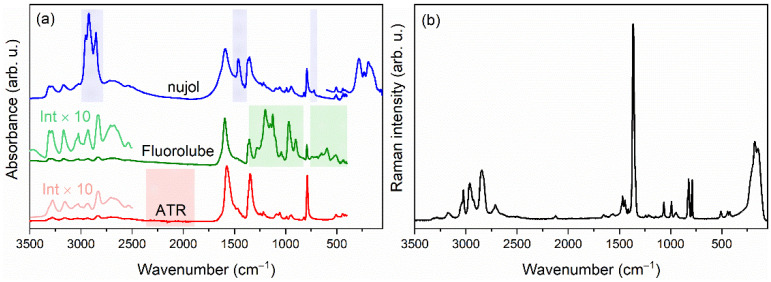
RT polycrystalline IR (**a**) and Raman (**b**) spectra of [DMHy]Mn(HCOO)_3_. The shaded fields correspond to regions where absorption bands of dielectric media (nujol, Fluorolube) or ATR crystal occur, and therefore, they are not analysed.

RT polycrystalline IR and Raman spectra are presented in Figure 2. The proposed assignment of the observed bands, based on comparative analysis and DFT data, is listed in Table 1. The assignment of the bands corresponding to formate ions is straightforward since internal vibrations of formate ions are commonly observed in narrow spectral ranges for other members of the large [A]Mn(HCOO)_3_ (A = protonated amine) family. For instance, the ν_1_, ν_2_, ν_3_, ν_4_, ν_5_ and ν_6_ modes were previously observed in the 2827–2888, 1352–1364, 789–805, 1562–1594, 1368–1387 and 1063–1071 cm^−1^ ranges, respectively, for analogues with A = Hy^+^ [18], MHy^+^ [20] and dimethylammonium cation [40]. Thus, we assign the IR and Raman bands of [DMHy]Mn(HCOO)_3_ observed in the 2826–2858, 1342–1352, 788–792, 1560–1593, 1363–1365 and 1056–1065 cm^−1^ ranges to ν_1_–ν_6_ vibrations, respectively. The assignment of the bands corresponding to DMHy^+^ in [DMHy]Mn(HCOO)_3_ crystal is based on our DFT calculations and previous ab initio calculations for DMHy molecule performed by Durig et al. [41]. The positions of ν_as_CH_3_, ν_s_CH_3_, ν_as_NH_2_ and ν_s_NH_2_ bands for [DMHy]Mn(HCOO)_3_ are in good agreement with the calculations. Weak bands between 3039 and 3054 cm^−1^, not present for DMHy, were assigned to νNH^+^. They are expected to be observed at lower wavenumbers than ν_as_NH_2_ and ν_s_NH_2_ bands because the –NH^+^ group is able to form stronger HBs [2]. The remaining stretching vibrations, ν_as_CNC, ν_s_CNC and νNN, are located in the 990–1002, 820–832 and 1089–1098 cm^−1^ range, respectively. The position of the ν_s_CNC bands is in good agreement with previous studies, i.e., this band was observed at 874 cm^−1^ for [MHy]Mn(HCOO)_3_ [20], 877 cm^−1^ for [MHy]Mn(H_2_POO)_3_ [42], 868 and 871 cm^−1^ for [MHy]PbBr_3_ [43] and 881 cm^−1^ for [MHy]PbCl_3_ [44]. The antisymmetric counterpart was previously observed at 1010 cm^−1^ for [MHy]Mn(H_2_POO)_3_ [42], 1004 cm^−1^ for [MHy]PbBr_3_ [43] and 1011 cm^−1^ for [MHy]PbCl_3_ [44]. For the [MHy]Mn(HCOO)_3_ crystal, this vibration was assigned to bands observed near 1092 cm^−1^ [20], but our DFT and previous ab initio calculations [41] showed that bands observed in the 1089–1098 cm^−1^ range for [DMHy]Mn(HCOO)_3_ originate from νNN. This mismatch relates to a different division into normal vibrations of the skeleton.

The assignment of bending vibrations of the skeleton is undoubted since they cover the spectral range free of any other vibrational bands. In this manner, bands in the 419–442 and 504–507 cm^−1^ were ascribed to δCNN and δCNC, respectively. The lack of δCNC bands for MHy^+^ analogues and the presence of δCNN ranging from 437–444 cm^−1^ [20,42,43] confirms this assignment.

The bending vibrations of the amino group are expected to be broader than bands corresponding to the methyl group, and therefore, we assign bands observed in the 1639–1654 range to δNH_2_. For other formate perovskites, they were observed in similar ranges, i.e., 1589–1654 cm^−1^ [20]. For non-formate analogues, the bending vibrations of protonated amino groups were observed 21–55 cm^−1^ lower than δNH_2_ [42,43]. Therefore, in the case of [MHy]Mn(H_2_POO)_3_, the δNH^+^ bands are expected to coincide with bands corresponding to the ν_4_ vibrations of formate ions. The distinction between the broad ρNH_2_, ωNH_2_ and τNH_2_ bands is more tentative in the literature, but our DFT results and previous ab initio calculations [41] are in good agreement. We found these vibrations in the 1342–1352, 937–958 and 219–282 cm^−1^ ranges, respectively. According to our DFT data, the γNH^+^ vibration is expected to contribute to bands at 1374 (1396) cm^−1^ and 1419 (1422) cm^−1^ in an anharmonic (scaled harmonic) model. Therefore, we assign weak and broad bands in the 1411–1439 cm^−1^ range to this vibration.

Vibrations of the methyl groups are expected to be less sensitive to the surroundings than vibrations of the amino groups. The bending modes (δ_as_CH_3_ and δ_s_CH_3_) can be assigned to the IR and Raman bands observed in the 1445–1480 and 1411–1439 cm^−1^ range, respectively. They were previously observed in similar ranges, i.e., 1454–1478 and 1421–1433 cm^−1^ [20,42]. The ρCH_3_ and τCH_3_ bands were found between 1098 and 1246 cm^−1^_,_ as well as between 219–282 cm^−1^, respectively. These ranges are in a good agreement with reported values for [MHy]Mn(HCOO)_3_ and [MHy]Mn(H_2_POO)_3_, namely 1092–1234 cm^−1^ (ρCH_3_) and 210–237 cm^−1^ (τCH_3_) [20,42]. Bands located below 240 cm^−1^ are assigned to lattice modes (Table 1).

### 3.5. Temperature-Dependent IR and Raman Spectra

**Figure 3:**
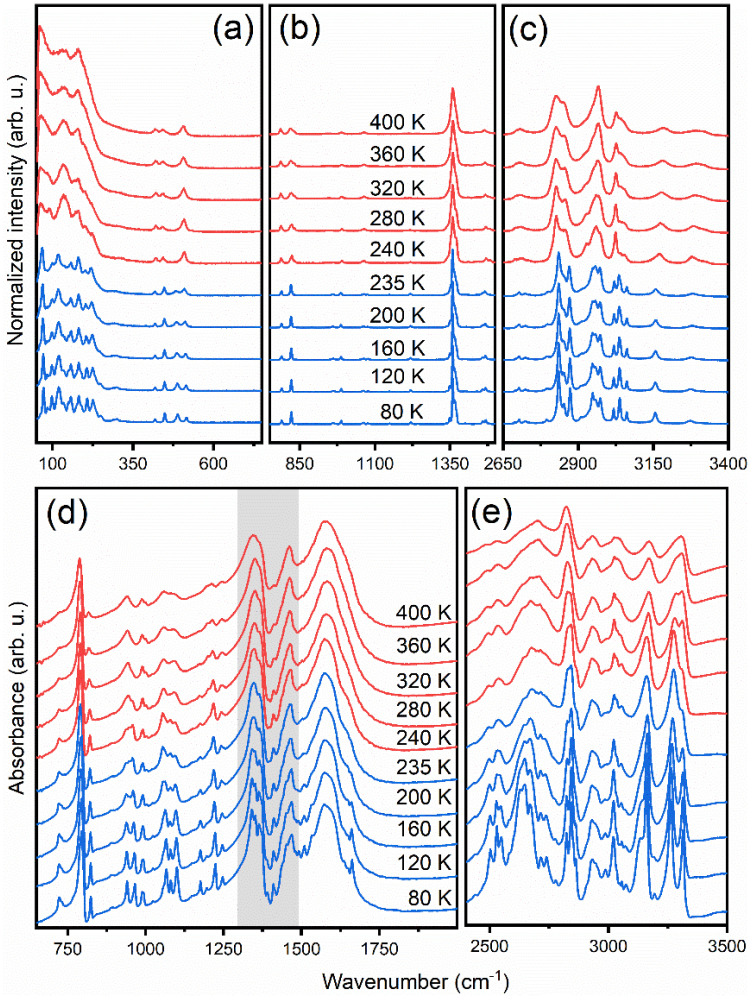
Temperature-dependent Raman spectra measured form single-crystal in the (**a**) 50–750 cm^−1^, (**b**) 750–1500 cm^−1^ and (**c**) 2650–3400 cm^−1^ ranges compared to temperature-dependent polycrystalline IR spectra measured in nujol (**d**) and Fluorolube (**e**). The grey range in (**d**) was not analysed due to the coexistence of bands corresponding to the nujol and sample.

The thermal evolution of Raman spectra (measured from single crystal) and polycrystalline IR spectra (measured as a suspension in Fluorolube and nujol) is presented in Figure 3. The observed wavenumbers at 80 and 300 K are listed in Table S4. To obtain more detailed information on the PT mechanism, the fitting of the IR and Raman spectra was conducted through the deconvolution of complex contours to Lorentzian curves.

#### 3.5.1. Internal Modes of Formate Ions

**Figure 4:**
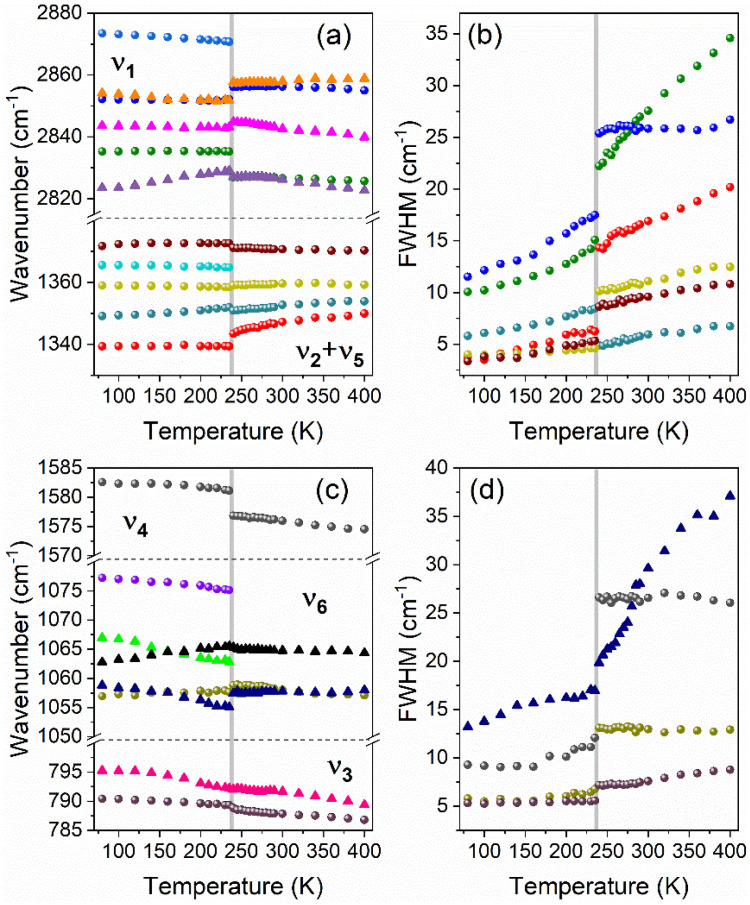
The temperature evolution of selected Raman (circles) and IR (triangles) wavenumbers (**a**,**c**) and bandwidths (**b**,**d**) of bands corresponding to the ν_1_, ν_2_ + ν_5_ (**a**,**b**) as well as to ν_3_, ν_4_ and ν_6_ (**b**,**d**) stretching vibrations of formate linkers. Horizontal dashed lines separate ranges of ν_1_–ν_6_ modes, vertical grey lines correspond to the PT temperature.

The resulting positions and full widths at half maximum (FWHM) of the IR and Raman bands corresponding to formate linkers are presented as a function of temperature in Figure 4. During the PT (on heating), the IR (Raman) bands above 2840 cm^−1^ corresponding to the ν_1_ mode exhibit upshifts by 1.8–6.5 cm^−1^ (3.9 cm^−1^), while bands below this limit downshift by 2.1 cm^−1^ (8.4 cm^−1^) (Figure 4a). The sensitivity of bands to the PT is also manifested as a significant broadening by 7.2–7.9 cm^−1^ (Figure 4b). Similar co-occurrence of positive and negative shifts is observed for the ν_5_ and ν_6_ bending vibrations. The hardening (softening) of the corresponding IR and Raman bands during the PT on heating does not exceed 2.4 cm^−1^ (1.4 cm^−1^). The corresponding increase in FWHMs for these modes ranges from 2.9 to 6.2 cm^−1^.

The ν_2_ and ν_4_ bands corresponding to stretching modes involving oxygen atoms exhibit downshifts by 1.1–4.2 cm^−1^ and upshift by 4.1 cm^−1^, respectively, at the PT temperature. The ν_4_ and one of the ν_2_ Raman bands exhibit strong broadening at the PT temperature upon heating, by 14.7 cm^−1^ and 7.7 cm^−1^, respectively. Interestingly, the second ν_2_ Raman band exhibits unusual narrowing by 3.5 cm^−1^.

The bending COC modes (ν_3_) seem to be less sensitive to the occurring structural transformation. Shifts of IR and Raman bands during the PT are weaker than 0.4 cm^−1^, and the broadening of the 790 cm^−1^ Raman band (1.6 cm^−1^) is the lowest among all ν_1_–ν_6_ bands. Selected detailed ranges of temperature-dependent Raman spectra corresponding to the ν_1_–ν_6_ formate anion internal modes are presented in Figure S3.

#### 3.5.2. Internal Modes of DMHy^+^ Cation

**Figure 5:**
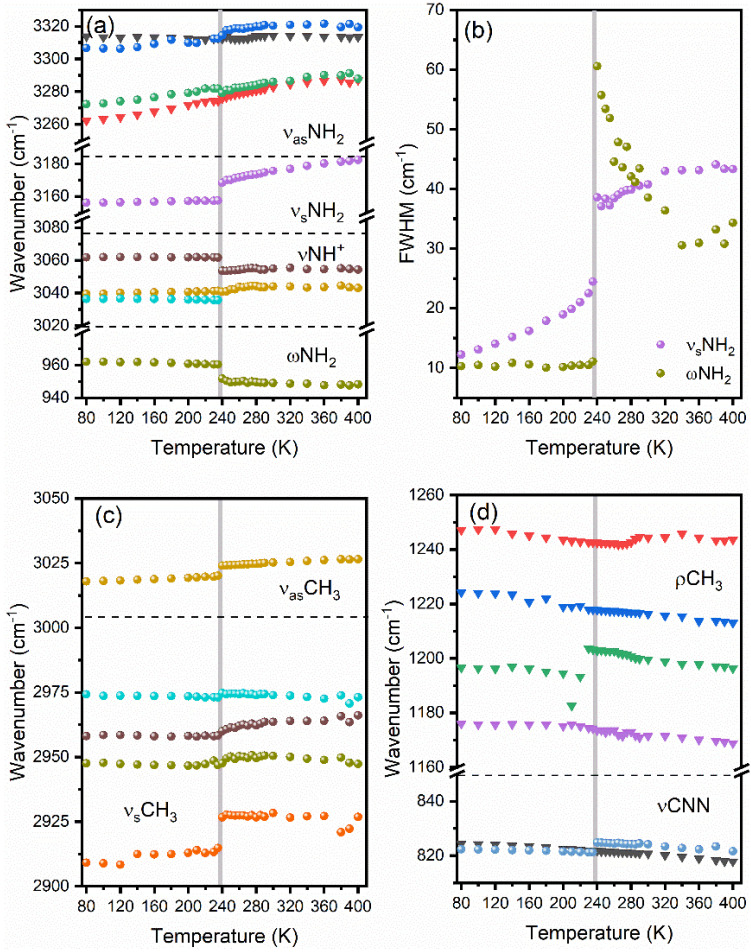
The thermal evolution of positions (**a**,**c**,**d**) and widths (**b**) of selected Raman (circles) and IR (triangles) bands corresponding to stretching and wagging vibrations of NH_2_ and NH^+^ groups (**a**,**b**), stretching vibrations of CH_3_ (**c**) and stretching vibrations of CNN skeleton and rocking vibration of CH_3_ group (**d**). Horizontal dashed lines separate ranges of different modes, and vertical grey lines correspond to the temperature of PT.

The results of fitting of IR and Raman bands corresponding to DMHy^+^ cation (positions and widths of the bands) are presented as a function of temperature in Figure 5. The details of IR and Raman spectra corresponding to DMHy^+^ vibrations are presented in Figures S4 and S5.

Two Raman bands observed above 3260 cm^−1^, corresponding to the ν_as_NH_2_ modes, exhibit weak shifts (less than 2.5 cm^−1^) at the PT temperature (Figure 5a). Almost no changes at the PT temperature are also observed for the IR counterparts. Raman bands corresponding to the ν_s_NH_2_ and ωNH_2_ modes are significantly more sensitive, i.e., they exhibit upshift by 10.8 cm^−1^ and downshift by 8.7 cm^−1^, respectively. They also disclose significant broadening by ca 14.1 cm^−1^ and 49.5 cm^−1^, respectively (Figure 5b). The shifts observed for the νNH^+^ modes are up to 8.0 cm^−1^.

Shifts observed for the Raman-active ν_as_CH_3_ and ν_s_CH_3_ modes are also strong, up to 12 cm^−1^. The rocking vibrations exhibit weaker changes and seem to be less affected by the PT. The only exception is the 1202 cm^−1^ mode (at 300 K), for which the upshift on heating is equal to 11.5 cm^−1^. The νCNN skeleton vibrations are weakly affected by the PT.

#### 3.5.3. Lattice Modes

**Figure 6:**
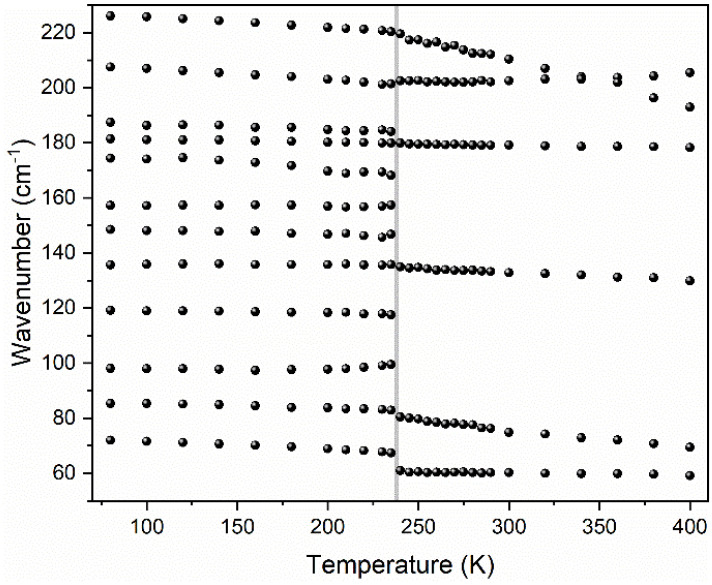
The temperature evolution of Raman lattice modes. The vertical grey line corresponds to the temperature of PT.

Figure 6 and Figure S6 show the temperature dependence of Raman bands observed in the 50–225 cm^−1^ range corresponding to the lattice modes. They exhibit the most significant changes during the PT. As one can see, after heating to 240 K, a few bands disappear. Furthermore, a large increase in bandwidth is observed (see Figure 6). Similar to the internal modes, they exhibit either up- or down-shifts. The strongest softening during the heating, by 6.7 cm^−1^, is observed for the lowest wavenumber mode located at 72 cm^−1^ (at 80 K). A weaker decrease in energy at the PT temperature, by 2.3, 0.9 and 0.7 cm^−1^, is observed for the 86, 136 and 227 cm^−1^ bands. The 180 cm^−1^ band is nearly insensitive to the change of temperature, while that at 208 cm^−1^ slightly hardens at 240 K, by 1.2 cm^−1^. Details of temperature-dependent Raman spectra corresponding to the range of lattice modes are presented in Figure S6.

## 4. Discussion

All observed dependencies show clear jumps at 240 K, evidencing the PT. A sudden character of these changes points to the first-order nature of this transformation. According to the selection rules, the *P*2_1_/*n*–*P*2_1_/*n* isosymmetric PT in [DMHy]Mn(HCOO)_3_ should not lead to any splitting or appearance of new bands for DMHy^+^ and HCOO^−^ ions. Nonetheless, some minor splitting of many internal modes below PT is evidenced. This effect is related to the thermal narrowing of closely lying and superimposed vibrational bands that become well separated at lower temperatures. On the other hand, factor group analysis showed that significant changes are expected for lattice modes with a strong contribution of T’(Mn^2+^). Indeed, pronounced changes upon cooling are observed for bands located below 225 cm^−1^. Our experiment shows that 6 of 12 Raman bands in this region disappear upon heating, which is in good agreement with our predictions. All 6 Raman-active T’(Mn^2+^) modes (3A_g_ + 3B_g_) in the LT phase become solely IR-active because of a change of symmetry to A_u_ and B_u_.

Such a strong splitting observed below 225 cm^−1^ confirms a strong deformation of the MnO_6_ octahedra in the LT phase. Since lattice modes are strongly coupled and involve librational and translational vibrations of all crystal units, this effect reflects also a strong deformation of the whole manganese–formate framework. This conclusion is further supported by the strong broadening of the ν_4_ mode corresponding to the antisymmetric stretching vibration of CO groups and the low sensitivity of the ν_3_ bending COC mode. The co-occurrence of up- and down-shits, observed in the same type of vibrational modes corresponding to the formate ion, suggests the presence of a few symmetrically independent formate linkers in the unit cell that have slightly different distortions before and after PT. The crystallographic data reported at 100 and 300 K are consistent with the spectroscopic data [2]. They show three different formate linkers in both LT and HT phases that have different susceptibility to structural distortions. At 300 K, two of them have increased C-O bonds by 1.4–1.8% and the COC angle by 2.6% in comparison to the LT phase, while the third linker has one weakly increased C-O distance (0.8%) and one strongly elongated (2.4%) C-O bond. The corresponding COC angle remains weakly affected by about 0.4% [2]. This phenomenon and co-occurring wide range of changes corresponding to Mn-O distances, from −0.36 to 0.19%, explain well the observed up- or down-shifts of vibrational bands corresponding to lattice modes and formate linkers.

Large broadening observed in the lattice mode region is also caused by the disordering of DMHy^+^ interplaying with the re-arrangement of HBs. This effect is most clearly visible for bands corresponding to both amino groups. A strong broadening at the PT temperature evidences the highly dynamic nature of this PT. Both IR and Raman bands corresponding to the νNH_2_ stretching modes experience shifts and sudden broadening at the PT during heating. In particular, IR and Raman bands corresponding to the ν_s_NH_2_ and ν_as_NH_2_ vibrations harden when going from the LT to the HT phase. This behaviour is strong evidence that the strength of HBs created by unprotonated amino groups is lower in the HT phase. In contrast to this behaviour, bands corresponding to the νNH^+^ modes soften during the PT on heating. This indicates that the HBs related to the protonated amino group become stronger in the HT phase, in agreement with the crystallographic data [2]. Furthermore, this behaviour proves that both types of amino groups play a crucial role in the PT mechanism. A significant broadening and change of shape of the νCH_3_ bands at the PT temperature can also be attributed to their disorder in the HT phase and their ability to form weak HBs.

The X-ray diffraction structural analysis showed that in the HT phase, the amino and methyl groups bonded to the middle N4 atom are disordered; however, one of the N4-(C|N) bonds is slightly shorter [2]. In the LT phase, the N-N bond is shorter compared to two N-C bonds, and the conformation of the skeleton is preserved [2]. We suppose, therefore, that most of the changes observed in the Raman and IR spectra are a consequence of cation ordering. The ordering of cations, along with the re-arrangement of HBs, because of tight confinement in the crystal void, forces the simultaneous deformation of the manganese–formate framework. The order-disorder mechanism is in good agreement with the high change of PT entropy observed for [DMHy]Mn(HCOO)_3_ [2].

## 5. Conclusions

We have studied phonon properties of manganese–formate framework templated by DMHy^+^ cations combining the DFT calculations and the temperature-dependent IR and Raman spectroscopy as a probe. We have presented selection rules and a correlation diagram for the LT and HT monoclinic (*P*2_1_/*n*) phases. We have proposed the assignment of the observed IR and Raman bands to the respective internal and external (lattice) vibrations based on the DFT calculations performed for the DMHy molecule and its single protonated cation and the comparative analysis. We have shown that some bands exhibit stronger anharmonic behaviours and are more sensitive to structural changes.

The detailed analysis of temperature-dependent Raman and IR studies allowed us to obtain deeper insight into the PT mechanism occurring in this hybrid perovskite. We have concluded that the unusual isosymmetric order-disorder phase transformation from one *P*2_1_/*n* to the second *P*2_1_/*n* phase occurring near 240 K has a highly dynamic nature because of the ordering of DMHy^+^ ions and re-arrangement of HBs. We have also proved that this transition has a first-order nature. The observed splitting of lattice modes below 240 K has been explained using selection rules that are slightly different for manganese ions in both phases.

Certainly, the mechanism of the PT involves the ordering of DMHy^+^ cations as suggested in the previous work. However, the analysis of the thermal evolution of particular bands revealed that the PT mechanism has a more complex nature and is a result of a few contributions. It involves the simultaneous ordering of the organic cations and re-arrangement of the HBs network, but without conformational change of the DMHy^+^ cations. This ordering and re-orientational motions, because of the tight confinement of the cations and steric hindrance, forces a strong deformation of the manganese–formate framework and MnO_6_ octahedra in the LT phase.

## Figures and Tables

**Table 1:** The tentative assignments of IR and Raman bands observed for polycrystalline [DMHy]Mn(HCOO)_3_.

Raman	IR (ATR)	IR (Nujol)	IR (Fluorolube)	Assignment
3286vw	3306sh, 3279w	3312w, 3283w	3310w, 3283w	ν_as_NH_2_
3173w	3157w	3171w	3170w	ν_s_NH_2_
3052sh, 3041w	3054vw, 3040vw	3054vw, 3039vw	3052sh, 3039sh	νNH^+^
3025m	3027vw	3025vw	3025w	ν_as_CH_3_
2968sh, 2963m, 2926m	2952sh, 2936vw	*	2953sh, 2931w	ν_s_CH_3_
2851sh, 2844w, 2831sh	2832w	*	2858w, 2843sh, 2826sh	ν_1_
2732sh, 2712w, 2684sh	2710w, 2676sh, 2534vw, 2498vw	2715w, 2674w, 2639sh, 2540vw, 2495vw	2717w, 2675vw, 2639sh, 2540vw, 2494vw	νNH_2_ + νNH^+^ + o + cb
1654vw	1639sh	1644sh	1642sh	δNH_2_
1578vw, 1560vw	1574vw	1591vs	1593vs	ν_4_ + δNH^+^
1480w, 1469w, 1445w	1478m, 1468m, 1447sh	*	1477w, 1467w, 1446vw	δ_as_CH_3_
1439sh, 1412w	1436vw, 1411vw	*	1436vw, 1411vw	δ_s_CH_3_ + γNH^+^
1365vs	1363sh	*	*	ν_5_
1345sh	1347vs	1352s, 1342sh	*	ν_2_ + ρNH_2_
1243vw, 1217vw, 1202vw, 1145vw	1244w, 1217w, 1202w, 1149vw	1246w, 1217w, 1201vw, 1146sh	*	ρCH_3_
1098vw	1089w	1093w	*	ρCH_3_ + νNN
1065w	1056w	1058w	*	ν_6_
1002vw, 991w	1002w, 991w	1002w, 990vw	*	ν_as_CNC
957w, 946w, 937vw	957w, 946vw	958w, 946w	*	ωNH_2_
832m, 823m	821w	820vw	820vw	ν_s_CNN
789m	788vs	792s	792m	ν_3_
507vw	506w	504w	*	δCNC
442vw, 421w	442sh, 421w	441w, 419w	*	δCNN
219sh		282s, 233m		τNH_2_ + τCH_3_ + lm
198sh, 177s, 144s		191s, 161sh		lm

Key: ν, stretching; δ, bending; ρ, rocking; γ, out-of-plane bending; ω, wagging; τ, twisting; vs, very strong; s, strong; m, medium; w, weak; vw, very weak; ν_1_–ν_6_, internal vibrations of formate ion (see description in text); *, regions of absorption related to the medium; o, overtones; cb, combinational bands; lm, lattice modes.

## Data Availability

The data presented in this study are available on request from the corresponding author.

## Supplementary Materials

The following are available online at https://www.mdpi.com/article/10.3390/ma14143984/s1, Table S1: Calculated and experimental bond angles and lengths of DMHy^+^ cation and DMHy molecule; Table S2: Optical modes in LT and HT phases; Table S3: Calculated PED and harmonic and anharmonic wavenumbers of DMHy^+^ and DMHy; Table S4: Experimental IR and Raman bands of LT and HT forms with the assignment; Figure S1: The numbering of atoms in DMHy^+^ and DMHy; Figure S2: Calculated IR and Raman spectra of DMHy^+^ and DMHy; Figure S3: Raman bands corresponding to vibrational modes of the formate anion; Figure S4: Details of temperature-dependent Raman spectra, corresponding to vibrational modes of DMHy^+^ cation; Figure S5: Details of temperature-dependent IR spectra, corresponding to vibrational modes of DMHy^+^ cation; Figure S6: Raman bands corresponding to lattice modes.
