# Supplementary material for: Mechanism of Unusual Isosymmetric Order-Disorder Phase Transition in [Dimethylhydrazinium]Mn(HCOO)3 Hybrid Perovskite Probed by Vibrational Spectroscopy
Source: Materials (Basel). 2021 Jul 16;14(14):3984. doi: 10.3390/ma14143984 (PMC8303174; doi:10.3390/ma14143984)
Supplement: Supplementary file 1 [file materials-14-03984-s001.zip › materials-1289167-supplementary.pdf]

materials

MDPI

Supplementary Material

Mechanism of Unusual Isosymmetric Order-Disorder Phase Transition in [Dimethylhydrazinium]Mn(HCOO)$_3$ Hybrid Perovskite Probed by Vibrational Spectroscopy

Jan Albert Zienkiewicz, Edyta Kucharska and Maciej Ptak

Table S1. Selected calculated and experimental bond lengths (Å) and bond angles (°) of dimethylhydrazinium cation ($DMHy^+$) compared to calculated values obtained for dimethylhydrazine molecule (DMHy).

DMHy$^+$ cation			DMHy molecule	
**Bond length / angle**	**Calculated**	**Experimental (300 K, 100 K) [1]**	**Bond length / angle**	**Calculated**
N1–N4	1.450	1.4268 (34), 1.4475 (34)	N1–N4	1.431
N1–H2	1.017	0.8899 (35)-0.8903 (27),	N1–H2	1.013
N1–H3	1.017	0.9852 (28)-1.0008 (25)	N1–H3	1.024
N4–C5	1.504	1.4766 (34), 1.4897 (42)	N4–C5	1.456
N4–C10	1.503	1.4722 (44), 1.4878 (40)	N4–C9	1.455
N4–H9	1.021	0.9781 (18), 0.9802 (25)		
C5–H6	1.086	0.9597 (30)-0.9601 (28),	C5–H6	1.090
C5–H7	1.087	0.9595 (31)-0.9603 (34)	C5–H7	1.091
C5–H8	1.086		C5–H8	1.105
C10–H10	1.087	0.9593 (38)-0.9604 (41),	C9–H10	1.103
C10–H11	1.086	0.9594 (27)-0.9603 (29)	C9–H11	1.090
C10–H12	1.085		C9–H12	1.087
N1–N4–C5	115.46	110.966 (218)-112.655 (238),	N1–N4–C5	112.51
N1–N4–C10	108.69	109.041 (215)-114.215 (227)	N1–N4–C9	108.53
C5–N4–C10	112.70	110.984 (219), 111.226 (233)	C5–N4–C9	111.99
N4–N1–H2	107.90	109.445 (311)-109.528 (319),	N4–N1–H2	107.38
N4–N1–H3	107.29	102.431 (219)-111.051 (226)	N4–N1–H3	109.99
N4–C5–H6	108.58	109.453 (246)-109.485 (246),	N4–C5–H6	109.61
N4–C5–H7	108.85	109.473 (246)-109.510 (322)	N4–C5–H7	109.88
N4–C5–H8	108.19		N4–C5–H8	112.49
N4–C10–H10	110.62	109.437 (281)-109.506 (258),	N4–C9–H10	108.47
N4–C10–H11	108.53	109.447 (258)-109.498 (254)	N4–C9–H11	109.42
N4–C10–H12	107.87		N4–C9–H12	108.94
C5–N4–H9	108.22	107.527 (186), 107.334 (236)		
N1–N4–H9	104.41	109.223 (218)-107.363 (227)		

Table S2. The correlation diagram showing the correspondence between the optical modes in the LT (HT) phases of [DMHy]Mn$(HCOO)_3$.

Ion	Vibration	Free ion symmetry	Site symmetry	Factor group symmetry LT (HT)
			**C_1=1 (C_i = $\bar{1}$)**	**C_{2h}=2/*m* (C_{2h}=2/*m*)**
**Mn^{2+}**	T′	-	3A (3A_u)	3A_g + 3A_u + 3B_g + 3B_u (6A_u + 6B_u)
		C_s	**C_1=1**	**C_{2h}=2/*m***
	$\nu_s NH_2$	A′	A	A_g + A_u + B_g + B_u
	$\nu_{as} NH_2$	A″	A	A_g + A_u + B_g + B_u
	δNH_2	A′	A	A_g + A_u + B_g + B_u
	ϱNH_2	A″	A	A_g + A_u + B_g + B_u
	τNH_2	A″	A	A_g + A_u + B_g + B_u
	ωNH_2	A′	A	A_g + A_u + B_g + B_u
	νNH	A′	A	A_g + A_u + B_g + B_u
	δNH	A′	A	A_g + A_u + B_g + B_u
**$DMHy^+$**	γNH	A″	A	A_g + A_u + B_g + B_u
	$\nu_s CH_3$	A′ + A″	2A	2A_g + 2A_u + 2B_g + 2B_u
	$\nu_{as} CH_3$	2A′ + 2A″	4A	4A_g + 4A_u + 4B_g + 4B_u
	$\delta_s CH_3$	A′ + A″	2A	2A_g + 2A_u + 2B_g + 2B_u
	$\delta_{as} CH_3$	2A′ + 2A″	4A	4A_g + 4A_u + 4B_g + 4B_u
	ϱCH_3	2A′ + 2A″	4A	4A_g + 4A_u + 4B_g + 4B_u
	τCH_3	A′ + A″	2A	2A_g + 2A_u + 2B_g + 2B_u
	νNN	A′	A	A_g + A_u + B_g + B_u
	νCN	A′ + A″	2A	2A_g + 2A_u + 2B_g + 2B_u
	δCNN	A′ + A″	2A	2A_g + 2A_u + 2B_g + 2B_u
	δCNC	A′	A	A_g + A_u + B_g + B_u
	T′	2A′ + A″	3A	3A_g + 3A_u + 3B_g + 3B_u
	L	A′+ 2A″	3A	3A_g + 3A_u + 3B_g + 3B_u
		**C_{2v}=*mm*2**	**C_1=1**	**C_{2h}=2/*m***
	ν_1	A_1	3A	3A_g + 3A_u + 3B_g + 3B_u
	ν_2	A_1	3A	3A_g + 3A_u + 3B_g + 3B_u
	ν_3	A_1	3A	3A_g + 3A_u + 3B_g + 3B_u
**$HCOO^-$**	ν_4	B_1	3A	3A_g + 3A_u + 3B_g + 3B_u
	ν_5	B_1	3A	3A_g + 3A_u + 3B_g + 3B_u
	ν_6	B_1	3A	3A_g + 3A_u + 3B_g + 3B_u
	T′	A_1 + B_1 + B_2	9A	9A_g + 9A_u + 9B_g + 9B_u
	L	A_2 + B_1 + B_2	9A	9A_g + 9A_u + 9B_g + 9B_u

Key: red: IR-active modes, blue: Raman-active modes, green: IR- and Raman-active modes.

Table S3. Calculated harmonic (ν_h) and anharmonic (ν_{ah}) wavenumbers for dimethylhydrazinium cation ($DMHy^+$) and dimethylhydrazine molecule (DMHy) as well as potential energy distribution (PED) of the respective predominant modes.

**DMHy⁺ Cation**				**DMHy Molecule**			
ν_h (cm^{-1})	ν_h* (cm^{-1})	ν_{ah} (cm^{-1})	PED (%)	ν_h (cm^{-1})	ν_h* (cm^{-1})	ν_{ah} (cm^{-1})	PED (%)
3556	3414	3389	$\nu_{as}NH_2$-100	3532	3391	3344	$\nu_{as}NH_2$-100
3470	3331	3363	ν_sNH_2-98	3345	3211	3148	ν_sNH_2-100
3445	3307	3288	νNH^+-95				
3190	3062	3047	$\nu_{as}CH_3$-100	3129	3004	2984	$\nu_{as}CH_3$-100
3174	3047	3030	$\nu_{as}CH_3$-99	3099	2975	2946	$\nu_{as}CH_3$-99
3172	3045	3028	$\nu_{as}CH_3$-100	3071	2948	2946	$\nu_{as}CH_3$-100
3165	3038	3022	$\nu_{as}CH_3$-100	3059	2937	2933	$\nu_{as}CH_3$-99
3083	2960	2983	ν_sCH_3-100	2920	2803	2746	ν_sCH_3-99
3074	2951	2971	ν_sCH_3-100	2905	2846	2723	ν_sCH_3-99
1685	1651	1658	δNH_2-99	1670	1637	1612	δNH_2-99
1515	1484	1467	$\delta_{as}CH_3$-70 + ρNH_2-14 + δNH^+-13	1513	1482	1468	$\delta_{as}CH_3$-98
1512	1482	1464	$\delta_{as}CH_3$-85	1497	1467	1462	$\delta_{as}CH_3$-98
1497	1467	1462	$\delta_{as}CH_3$-66 + δNH^+-24				
1492	1462	1442	$\delta_{as}CH_3$-79 + δNH^+-16	1495	1466	1449	$\delta_{as}CH_3$-98
1481	1451	1442	$\delta_{as}CH_3$-79 + δNH^+-16	1483	1453	1448	$\delta_{as}CH_3$-99
1473	1444	1438	δ_sCH_3-96	1463	1434	1422	δ_sCH_3-98
1452	1422	1419	δ_sCH_3-89 + γNH^+-11	1439	1410	1414	δ_sCH_3-100
1424	1396	1374	γNH^+-66 + δ_sCH_3-34				
1359	1332	1321	ρCH_3-43 + ρNH_2-40 + δNH^+-16	1340	1314	1296	ρNH_2-77 + νCN-17
1255	1230	1212	ρCH_3-81 + νNN-10	1284	1258	1245	ρCH_3-71 + ρNH_2-29
1218	1194	1178	ρCH_3-71 + ωNH_2-17	1241	1217	1202	ρCH_3-47 + ωNH_2-47
1138	1116	1112	ρCH_3-74 + ρNH_2-19	1167	1144	1137	ρCH_3-81 + ρNH_2-11
1060	1039	1026	ρCH_3-48 + ωNH_2-45	1115	1093	1087	ρCH_3-98
1050	1029	1025	ρCH_3-67 + ωNH_2-22 + γNH^+-10	1080	1058	1046	ρCH_3-50 + ωNH_2-30 + νNN-20
958	938	923	νCN-95	1029	1008	1005	ρCH_3-53 + $\nu_{as}CNC$-45
915	897	867	νNN-41 + νCN-31 + ωNH_2-26	950	931	898	ωNH_2-49 + $\nu_{as}CNC$-28 + νNN-19
793	777	766	νCN-64 + νNN-30	816	799	788	νNN-44 + ν_sCNC-58
475	466	467	δCNN-89	464	455	454	δCNC-96
426	417	425	δCNN-90	428	420	429	δCNC-99
391	384	364	δCNC-63 + δCNN-27	399	391	371	δCNN-72 + δCNC-27
264	259	231	τCH_3-99	300	294	318	τNH_2-98
262	257	216	τNH_2-99	273	268	299	τCH_3-100
216	212	200	τCH_3-100	247	242	287	τCH_3-98

* scaling factor, 0.9800 (0-2499 cm^{-1}), 0.96 (2500-3600 cm^{-1}); key: ν, stretching; δ, bending; ρ, rocking; γ, out-of-plane bending; ω, wagging; τ, twisting

Table S4. The comparison of IR (polycrystalline) and Raman (single crystal) bands observed at 80 and 300 K for $[DMHy]Mn(HCOO)_3$.

Raman (80 K)	Raman (300 K)	IR (80 K)	IR (300 K)	Assignment
3307sh, 3272vw	3320sh, 3286vw	3352vw, 3314w, 3262m, 3227sh	3312w, 3283w	$\nu_{as}NH_2$
3156w	3176vw	3195vw, 3161m, 3135w	3171w	$\nu_{s}NH_2$
3062w, 3039sh, 3036m	3055sh, 3044w	3074vw, 3057vw, 3046vw	3054sh, 3039sh	νNH^+
3018m	3025m	3035sh, 3021w	3025w	$\nu_{as}CH_3$
2974m, 2958sh, 2948m, 2909sh	2974sh, 2964m, 2950sh, 2928m	2987w[a], 2947w[a], 2931w[a]	2953sh[a], 2931w[a]	$\nu_{s}CH_3$
2873s, 2852m, 2835s	2856w, 2827m	2863w[a], 2854sh[a], 2844w, 2824w[a]	2858sh[a], 2843sh[a], 2826w[a]	ν_1
2795w, 2724vw, 2703w, 2690vw	2710vw, 2692vw	2778vw, 2740w, 2716w, 2670w, 2648m, 2628m, 2602sh, 2584sh, 2550w, 2531w, 2504w	2730sh, 2715w, 2674w, 2639sh, 2540vw, 2495vw	$\nu NH_2+\nu NH^+$+o+cb
1648vw	1654vw	1661w	1648sh	δNH_2
1583vw	1576vw	1575vs, 1536sh, 1510m	1581vs	$\nu_4+\delta NH^+$
1503vw, 1481sh, 1469w, 1459w	1482sh, 1469vw	*	*	$\delta_{as}CH_3$
1440vw, 1401vw	1434vw, 1410vw	*	*	$\delta_{s}CH_3+\gamma NH^+$
1372sh, 1366s, 1359vs	1371sh, 1360s,	*	*	ν_5
1349m, 1340vw	1353sh	*	*	$\nu_2+\varrho NH_2$
1243vw, 1220w, 1149w	1243vw, 1217vw, 1143vw	1247w, 1224w, 1197vw, 1176w, 1151vw	1244w, 1216w, 1200w, 1171vw	ϱCH_3
1084sh, 1077w	1097vw	1100w, 1081w	1093w	$\rho CH_3+\nu NN$
1063w, 1057sh	1065w, 1058sh	1067w 1059sh	1058w	ν_6
		994 w, 989 w	1002 w, 990 vw	$\nu_{as}CNC$
962w	949vw	966w, 941w	958w, 946w	ωNH_2
839vw, 822m	833vw, 824w	825w	820vw	$\nu_{s}CNN$
790w	788wm	800sh, 795s, 767sh	792s	ν_3
516vw, 489vw	507vw			δCNC
449 vw, 419 vw	443 vw, 420 vw			δCNN
301vw, 290vw, 251vw, 226w, 208w	314vw, 210sh, 203sh			$\tau NH_2+\tau CH_3$ + lm
187sh, 181w, 174sh, 157w, 148w, 136w, 119w, 98w, 85w, 72w	179w, 133w, 75sh, 60w			lm

Key: ν, stretching; δ, bending; ρ, rocking; γ, out-of-plane bending; ω, wagging; τ, twisting; vs, very strong; s, strong; m, medium; w, weak; vw, very weak; ν_1-ν_6, internal vibrations of formate ion (see description in text); *, regions of absorption related to the medium; o, overtones; cb, combinational bands; lm, lattice modes; [a] in Fluorolube.

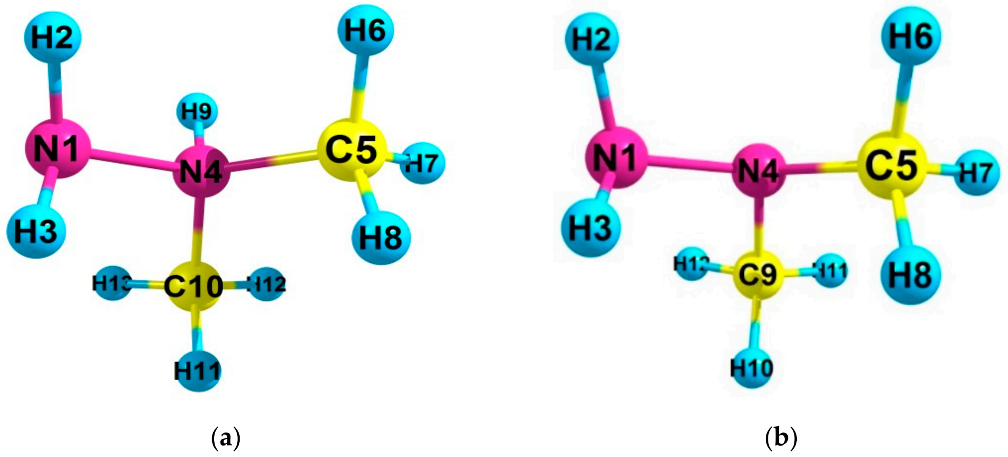

Figure S1. The numbering of atoms in the (**a**) dimethylhydrazinium cation ($DMHy^+$) and (**b**) dimethylhydrazine molecule (DMHy).

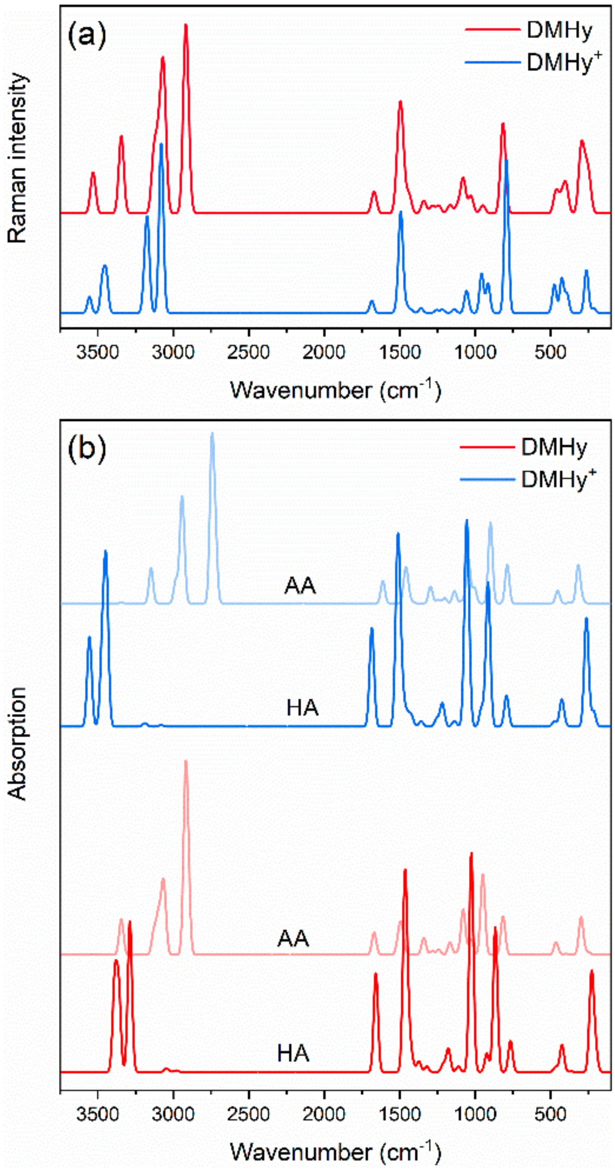

Figure S2. The comparison of calculated Raman spectra (**a**) in harmonic approximation and IR spectra (**b**) of dimethylhydrazinium cation (DMHy+) and dimethylhydrazine molecule (DMHy) in the harmonic (HA) and anharmonic approximation (AA).

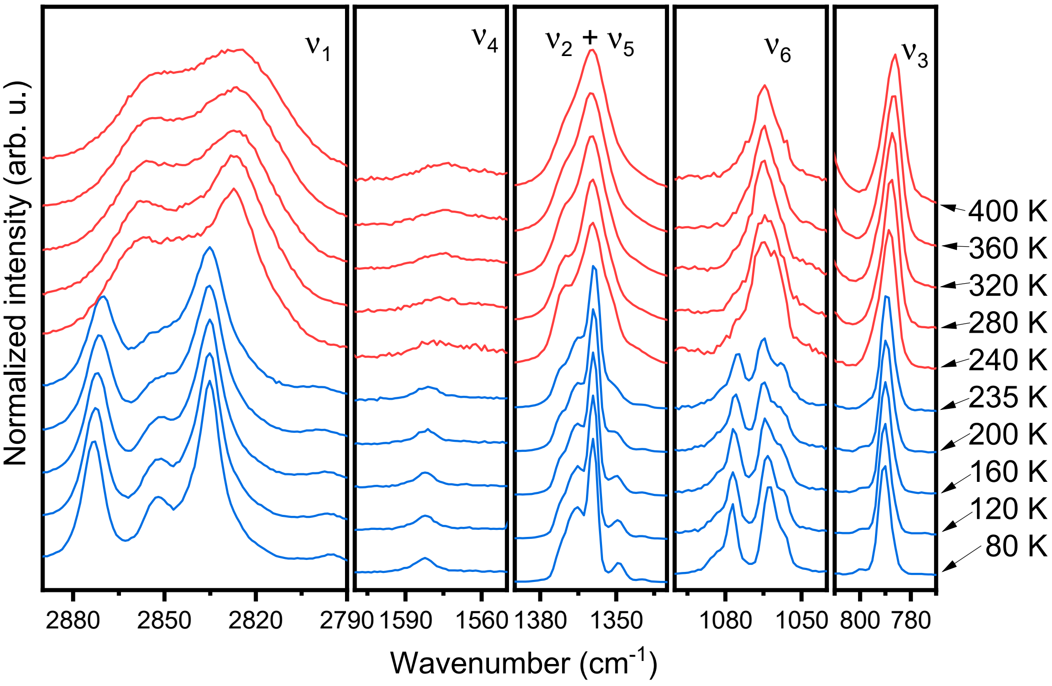

Figure S3. Raman bands corresponding to vibrational modes of the formate anion.

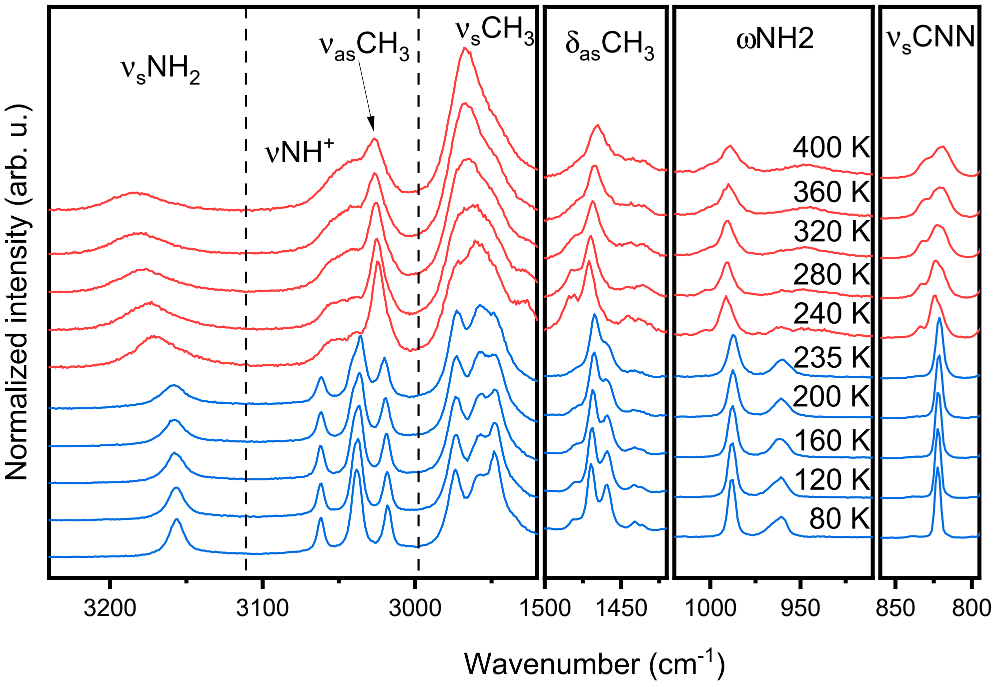

Figure S4. Details of temperature-dependent Raman spectra, corresponding to vibrational modes of DMHy+ cation.

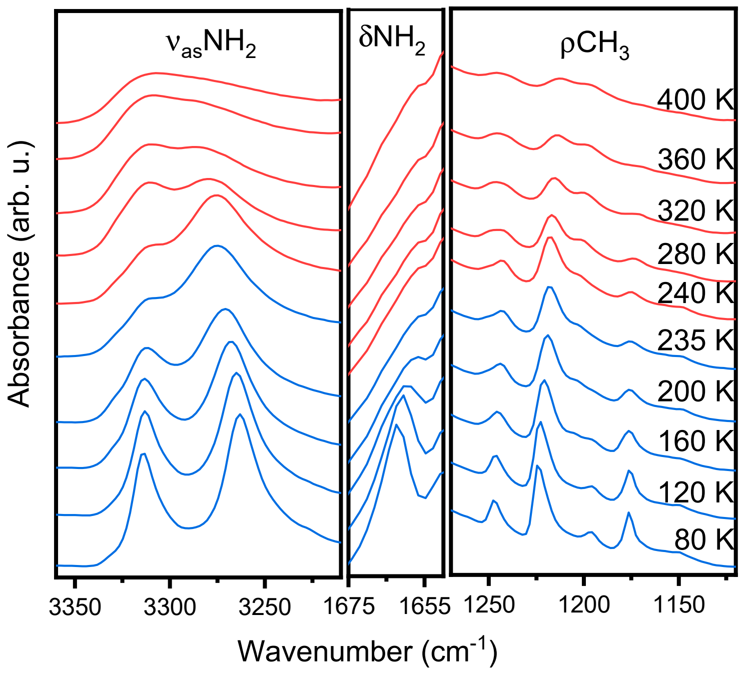

Figure S5. Details of temperature-dependent IR spectra, corresponding to vibrational modes of $DMHy^+$ cation.

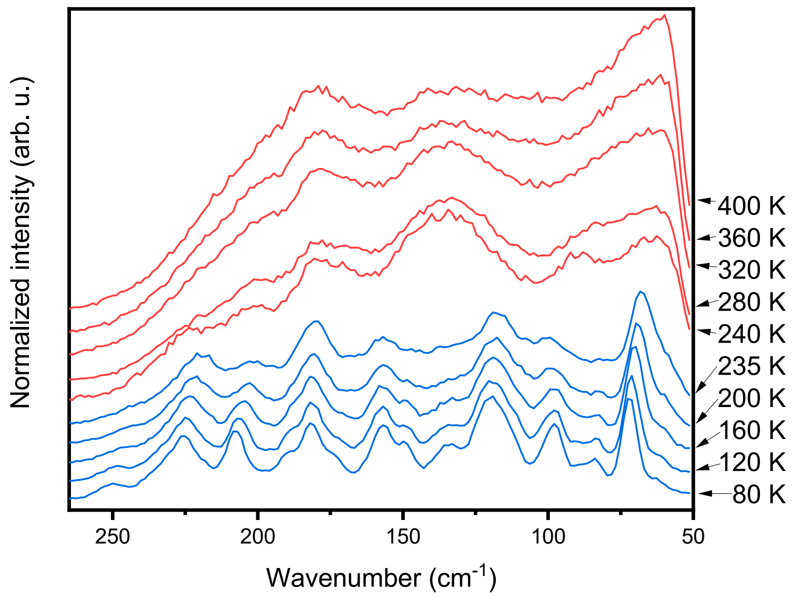

Figure S6. Raman bands corresponding to lattice modes.

Reference

1. Zienkiewicz, J.A.; Kowalska, D.A.; Fedoruk, K.; Stefański, M.; Pikul, A.; Ptak, M. Unusual isosymmetric order-disorder phase transition in the new perovskite-type dimethylhydrazinium manganese formate exhibiting ferrimagnetic and photoluminescent properties. *J. Mater. Chem. C* **2021**, 9, 6841-6851, https://doi.org/10.1039/D1TC01014J
